# Concordance between the Clinical Definition of Polypathological Patient versus Automated Detection by Means of Combined Identification through ICD-9-CM Codes

**DOI:** 10.3390/jcm8050613

**Published:** 2019-05-06

**Authors:** Juan Gómez-Salgado, Máximo Bernabeu-Wittel, Carmen Aguilera-González, Juan Antonio Goicoechea-Salazar, Daniel Larrocha, María Dolores Nieto-Martín, Lourdes Moreno-Gaviño, Manuel Ollero-Baturone

**Affiliations:** 1 Department of Sociology, Social Work and Public Health, Universidad de Huelva, 21007 Huelva, Spain; salgado@uhu.es; 2 Safety and Health Posgrade Programme, Universidad Espíritu Santo, Guayaquil 092301, Ecuador; 3 Department of Internal Medicine, Virgen del Rocío University Hospital, University of Seville, 41013 Seville, Spain; mcaguilerag@gmail.com (C.A.-G.); lonietoma@gmail.com (M.D.N.-M.); morenogavinol@gmail.com (L.M.-G.); mollero1@us.es (M.O.-B.); 4 Healthcare Product Service, Andalusian Health Service, 41071 Seville, Spain; jantonio.goicoechea@juntadeandalucia.es (J.A.G.-S.); Daniel.larrocha.sspa@juntadeandalucia.es (D.L.)

**Keywords:** cost effectiveness, multiple chronic conditions, population health management, patient safety, multimorbidity, polypathological patient, ICD-9-CM

## Abstract

It is unknown whether the digital application of automated ICD-9-CM codes recorded in the medical history are useful for a first screening in the detection of polypathological patients. In this study, the objective was to identify the degree of intra- and inter-observer concordance in the identification of in-patient polypathological patients between the standard clinical identification method and a new automatic method, using the basic minimum data set of ICD-9-CM codes in the digital medical history. For this, a cross-sectional multicenter study with 1518 administratively discharged patients from Andalusian hospitals during the period of 2013–2014 has been carried out. For the concordance between the clinical definition of a polypathological patient and the polypathological patient classification according to ICD-9-CM coding, a 0.661 kappa was obtained (95% confidence interval (CI); 0.622–0.701) with *p* < 0.0001. The intraclass correlation coefficient between both methods for the number of polypathological patient categories was 0.745 (95% CI; 0.721–0.768; *p* < 0.0001). The values of sensitivity, specificity, positive-, and negative predictive values of the automated detection using ICD-9-CM coding were 78%, 88%, 78%, and 88%, respectively. As conclusion, the automatic identification of polypathological patients by detecting ICD-9-CM codes is useful as a screening method for in-hospital patients.

## 1. Introduction

In the last third of the 20th century, there has been a demographic revolution among developed countries, as evidenced by a high proportion of the elderly and a parallel increase in life expectancy at birth. In Europe, the population aged 65 years or over is projected to increase from 15% in 2000 to about 30% by 2050, and, to this date, the population over the age of 80 is expected to triple, reaching 10% [1].

As people become older, the weight of acute processes on morbidity and mortality decreases, so chronic processes are becoming more frequent [2]. Chronic diseases are very directly related with multimorbidity, involving comprehensive care both at a hospital level and at a primary care level [3]. In Europe, chronic diseases account for a significant proportion (77%) of the total burden of diseases and are responsible for 86% of all deaths [4]. In the case of Spain, chronic diseases represented 90% of the causes of death in 2005, while they currently represent around 92%. On the other hand, chronically-ill patients consume 70% of health expenditure, 60% of hospitalizations, and 70%–80% of primary care consultations [5]. There is a tendency towards the coexistence of more than one chronic disease in the same person as age increases, which multiplies expenditure by six, compared with the patients with just one chronic illness, and by four or twelve times in comparison with patients of a lower age [6,7].

A polypathological patient (PP) is a patient with chronic diseases included in two or more different predefined categories, for which it is difficult to establish the protagonism of any of the diseases, as they are generally equivalently complex and with a similar potential for destabilization, management difficulties, and mutual interrelations [8]. These characteristics define the following profile: elderly population, with more functional limitations and higher mortality rates [9]. This implies a greater use of health care resources, a poorer quality of life, and high rates of adverse effects [10].

The prevalence of PP in internal medicine services has proven to be higher than 30% and close to 60% in services oriented to chronic patients [11]. In primary care, this collective means this is less than 1.4% of the general population and around 5% of the population over 64 years of age. PP are characterized by a high clinical complexity, with one-third of patients presenting three or more defining categories of PP, and 80% of them showing the presence of chronic diseases not included in the definition, as well as with a Charlson index score higher than 3.45. Because of this complexity, around 94% of the PP are polymedicated, with a mean of eight chronically prescribed medications per patient and a high prevalence of drugs interactions [12,13]. It has also been observed that this population is at risk for developing disability and dependency. Thus, the percentage of patients with functional impairment, measured through the Barthel index, is highly significant—more than a third (34%) in primary care [14]. More than 60% require the aid of a caregiver, and 40% of these caregivers show signs of overburden [15], mostly related to socio-family circumstances [16].

The adequacy of health services to the new chronicity reality is an important change that will require both proper strategic management from managing institutions, and professional involvement on the part of clinicians [17].

In recent years, special interest has been shown to find a prognostic rate of morbidity and mortality for PP [6]. This concept has already been known for a long time, and effort has been made to define it through instruments such as the widely used Charlson comorbidity index [18]. There has also been a tendency towards introducing stratification scales with prognosis interest (Chronic Illness Resources Survey scale (CIRS) [19], Index of Coexisting Disease (ICED index) [20], Kaplan–Feinstein index [21], etc.), which have been the subject of recent revisions [5]. The drawback of such indexes is that they do not value functional deterioration, or that it is often difficult to decide on which of the multiple processes the patients present is mainly responsible for their deterioration [18].

Currently, clinical evaluation is required, and there is no automatic detection tool based on clinical-administrative data. In a study developed by Wang H.E. et al., the authors tested the ability of the REasons for Geographic and Racial Differences in Stroke-Severe Sepsis Risk Score (REGARDS-SSRS) to predict 10-year severe sepsis risk in separate cohorts of community-dwelling adults. They based their study on the fact that there are no validated systems for characterizing the long-term risk of severe sepsis in community-dwelling adults, concluding that the REGARDS-SRSS may potentially play a role in identifying community-dwelling adults at high severe sepsis risk [22]. A possible solution to this issue is the use of the minimum basic data set (MBDS) and the application of the international classification of diseases criteria, revised and clinically modified (ICD-9-CM), in the diagnosis of a pathology, thus implying a more immediate identification. The MBDS is a clinical and administrative database that encompasses the computerized medical history, and that is obtained at patient discharge. This information is collected for each episode of hospital assistance, defined as the period between the hospital admission and the patient discharge. The clinical coding of the diagnoses and therapeutic and surgical procedures contained in the discharge medical report is carried out according to the ICD-9-CM, published by the World Health Organization (WHO), which is reviewed and updated every two years [23].

The MBDS contains highly valuable information about the health reality of a population. In addition to collecting the usual demographic data (age, sex, place of residence, and financing), it also records the diagnosis that motivated the admission (main diagnosis), the risk factors, comorbidities and complications that the patient presents during the admission (secondary diagnoses), some relevant diagnostic techniques, and the therapeutic interventions, especially of surgical type, that have been used to treat the patient (procedures). Other studies have previously relied on the automated identification of diseases, such as the one developed by Harnod T. et al. in Taiwan. In their population-based cohort study on 7872 patients, they analyzed whether hysterectomies were associated with an increased risk of depression, using the National Health Insurance Research Database of Taiwan for its development. The outcomes revealed that hysterectomy would be a predisposing factor for an increased risk of subsequent depression [24].

The identification of patients based on the knowledge, techniques, and experience of clinicians allows for health professionals to accurately identify current complex patients, but cannot really predict who will be at high risk in a future time frame. The identification of patients by clinical criteria has the disadvantage of the variability of criteria and the possibility of errors, which compromises the reliability of the method. The use of a combination of predictive information tools and clinical criteria could increase the degree of confidence in the classification of PP [25].

Regarding the PP identification by clinical criteria, clinicians rely on the PP defining criteria published by the Ministry of Health of Andalusia in 2007 [26]. According to these, a PP is considered to suffer chronic diseases framed within at least two of the eight clinical categories that are defined by diseases listed within one or more ICD-9-CM codes, as shown in Table 1.

Currently, this diagnosis is carried out by applying the established clinical criteria. In this work, as described above, the development of a population screening method by means of the identification of polypathological patients through the use of an administrative computer database, such as the MBDS and its ICD-9-CM coding, is proposed.

Our objective, therefore, has been to know the degree of intra- and inter-observer concordance of the identification of in-patient PP between two different methods—the standard clinical identification method and a new automatic method using ICD-9-CM coding

## 2. Experimental Section

This is a multicenter cross-sectional study, including all patients administratively discharged in the Andalusian MBDS, which included the discharges and deaths during the hospitalization episode in the period of 2013–2014.

### 2.1. Study Population

All of the hospitalized patients in different health areas of Andalusian public hospitals that had implemented the digital medical history, during the period of 2013–2014.

### 2.2. Inclusion Criteria

- Administrative discharge in the Andalusian MBDS of the index episode in the services of internal medicine, infectious diseases, digestive, cardiology, pulmonary, neurology, endocrine, hematology, rheumatology, and nephrology specialties.
- Possibility of access to the digital medical history and discharge of the index episode.
- Patients >18 years.

### 2.3. Exclusion Criteria

- Not meeting the inclusion criteria.
- Discharge encoded by childbirth and/or pregnancy pathology, from pediatric and surgical areas.

### 2.4. Sample

The sample size required was 1300 for a kappa coefficient of 0.7, according to the clinician classification of 40% of the sample as being polypathological and the computer application classification of 50% of the sample; for the detection of a sensitivity and specificity above 80% of the new PP detection method; for a power of 80%, a confidence level of 99%, and a precision of 0.05. Assuming a 10% loss (incomplete records), the final sample size was established as 1518. The study sample was recruited through a stratified probabilistic sampling according to the level of the hospitals and according to the volume of discharge reports in the years 2013 and 2014.

### 2.5. Variables

The main independent variable was the clinical cataloguing of the patient as polypathological, by three independent researchers who are experts in comorbidity and polypathology, done according to the criteria established by the group of experts of the Ministry of Health. The patients were considered PP if after reviewing the clinical documentation of each episode, two or three of the researchers identified them as PP. 

The main dependent variable was the cataloguing of the patient as polypathological by the ICD-9-CM coding system of the Anadalisian MBDS, as detailed in the definitions section. The patients were considered PP if they met two or more categories of the definition, each clinical category being defined as the completion of some disease that corresponds to the predetermined ICD-9-CM code(s) for each of the categories. This cataloguing was done automatically through the Andalusian MBDS program.

In addition, the sociodemographic variables were calculated for the description of the sample.

### 2.6. Statistical Analysis

The descriptive analysis of the quantitative variables was carried out by means of the determination of robust central values and dispersion values, depending on the distribution of each of them. The Kolmogorov–Smirnov test was used for the determination of the distribution, and the qualitative variables were described by percentages. For the concordance between the two methods of PP definition, the kappa index was used, both globally and differentiating by categories. Also, among those listed by both methods as PP (positive concordance), the concordance was analyzed by inclusion categories to check whether the cataloguing was performed accordingly, with the same defining categories. To complete the concordance analysis, the overall percentage of agreement between the two methods and among the researchers was calculated. For the concordance between the number of PP inclusion categories in both methods, the intraclass correlation coefficient was used.

Additionally, the sensitivity, specificity, and positive- and negative predictive values of the automatic identification of PP were calculated using the automated method based on ICD-9 codes, assuming the clinical cataloguing as the absolute truth criterion. Subsequently, a bivariate analysis of the factors associated with a greater concordance between the two methods was carried out. 

Finally, a multivariate logistic regression model was constructed from the predictive factors of the univariate analysis, and those that were additionally considered clinically relevant. It was developed backwards and step by step in order to determine those factors that were independently associated with the greatest concordance between the defining methods of PP.

The statistical significance threshold was set as <0.05 for *p*-values. All of the calculations were carried out through the statistical package Statistical Package for the Social Sciences (SPSS version 21.0, SPSS Inc., Chicago, IL, USA).

### 2.7. Ethical Aspects

The study was carried out following the "Ethical Principles for Medical Research with Human Beings", collected in the latest version of the Helsinki Declaration (Edinburgh Version, October 2000), for the development and monitoring of this clinical research. It has been subjected to the review and authorization of the Virgen Del Rocío University Hospital Ethics Committee of Research with code 2014PI/024, obtaining its approval. The data obtained during the study were treated according to law 5/1999 and applicable regulations. Written informed consent was requested from patients prior to participation. 

## 3. Results

A total of 1518 patients from 17 hospitals were included; 46.44% (*n* = 705) came from regional hospitals (third-level hospitals), 32.08% (*n* = 487) from specialty hospitals (second-level hospitals), and 21.47% (*n* = 326) from county hospitals (first-rate hospitals). Of the 1518 patients included, 851 (56.1%) were men and 667 (43.9%) were women. The median age was 71 years (P25 = 58; P75 = 80).

As for the number of ICD-9-CM diagnoses grouped at discharge, 13.6% (*n* = 207) had 15 diagnoses, 8.8% (*n* = 133) had 9, and 8.5% (*n* = 129) had 8. The median was nine diagnoses (P25 = 6; P75 = 12).

**Figure 1:**
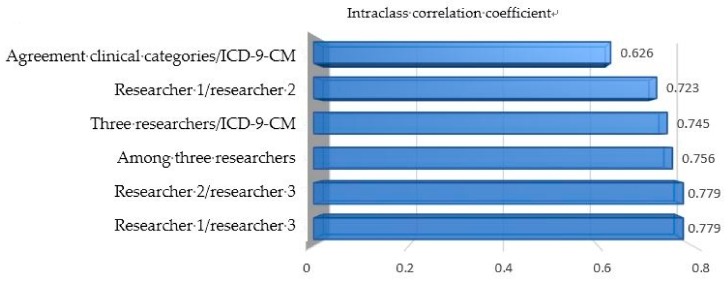
Intraclass correlation coefficient between the number of polypathological patient (PP) categories identified through clinical identification and through automated identification with ICD-9-CM coding.

It is noted that the concordance of the PP classification according to the clinical agreement between two or more researchers, and the PP classification according to the ICD-9-CM coding was good. A 0.661 kappa (95% CI; 0.622–0.701) with statistical significance (*p* < 0.0001) was obtained. Regarding the proportion of global agreement between the observers, a result of 0.844 (95% CI; 0.825–0.862) was obtained. According to the intraclass correlation coefficient of the number of defining categories detected by both methods, a notable result from the three researchers is obtained, as well as a low one from the clinical agreement of two or more researchers and the ICD-9-CM coding, as seen in Figure 1.

The concordance by clinical categories according to the clinical agreement and ICD-9-CM was very good in all of the categories, except for the E category, where it was moderate, and in the H category, where it was low. The category analysis is collected in Table 2. 

The multivariate analysis of the factors associated with the correct identification of PP using ICD-9-CM coding (with respect to the gold standard clinical identification method) is detailed in Table 3.

In the sensitivity (S), specificity (Sp), positive predictive value (PPV), and negative predictive value (NPV) analysis of the PP detection method based on ICD-9-CM coding, and the PP classification according to the clinical agreement of two or more researchers, was established as a benchmark or absolute truth. The calculation was carried out through the ICD-9-CM classification of PP with and without the H category. The results are described in Table 4. As can be seen, the results obtained were similar.

## 4. Discussion

The ICD-9-CM coding of the different pathologies is a novel subject that has been studied little so far. However, there are some studies where ICD-9-CM coding is generally understood as a homogenisation of the clinical language, allowing for the real results of the daily medical activity. At the national level, there are works based on the coding of different pathologies and symptoms, such as pancreatitis [27], thromboembolic diseases [28], diabetes mellitus [29], acute nosocomial gastroenteritis by rotavirus [30], chronic pain [31], and heart failure [32]. As it is known, the minimum basic data set (MBDS) incorporates ICD-9-CM for the diagnoses and therapeutic procedures of any specialty, so some works have tried to demonstrate the quality of the surgical procedures’ codification [33], as well as of hip and knee arthroplasty [34]. In addition, the implementation of this coding and its acceptance by health professionals will improve the management of health services so that they are able to carry out varied studies on this issue [35].

At an international level, some studies are worth mentioning that explore the possibility of using ICD-9-CM coding for the identification of comorbidities [36], chronic diseases [37], acute venous thrombosis [38], visits to emergencies related to acetaminophen [39], hypoglycemia visits [40], diabetic foot infections [41], or for the evaluation of morbidity [42].

The use of ICD-9-CM has been oriented, since its creation, towards hospital use. However, its integration into primary care is currently been sought after, by encoding the reasons for consultation and the diagnosed pathologies [43]. If there is a correct identification of polypathological patients through ICD-9-CM, and the family doctor and specialists in charge of the patient are subsequently notified, a correct continuity of care and communication between the different areas would be possible. Clinically speaking, a better control of the patient would be favored, which would lead to a decrease in the number of admissions of such patients.

It should be noted that there is little literature in which ICD-9-CM codes are used for the identification of PP, so contrasting the concordance results of our work with others, both nationally and internationally, has been impossible. At a national level, the initial Andalusian assistance process [26] includes a first attempt to adapt ICD-9-CM coding to the definitive clinical criteria of PP. It was Fernandez Miera [44] who carried out a descriptive study of the minimum basic data set (MBDS) and the identification of PP through ICD-9-CM coding, following the functional definition by the process of integrated assistance to the PP care of the Ministry of Health of the Government of Andalusia, in 2002. Being descriptive, the only aim was the identification of patients, not the concordance between the functional definition and ICD-9-CM coding. No further study has been done in which ICD-9-CM codes were used for the identification of PP.

The concordance does not evaluate the validity or certainty of any observation in relation to a given reference standard, but rather it evaluates how coherent the observations are on the same phenomenon among them. In these cases, the studies are considered to evaluate the consistency between the methods or instruments. In studies where one of the new methods or instruments is compared to the standard gold or benchmark method, the method’s conformity to the reference pattern, also called validity or operating performance of a diagnostic test [45], is assessed.

The concordance between the PP identification, through ICD-9-CM coding, obtained a good and concordance strength with respect to clinical identification. These results confirm the possibility of using the automatic identification of polypathological patients through ICD-9-CM coding as a population screening method. The creation of an automatic identification system for polypathological patients will allow for creating warning systems that contribute to planning the assistance these patients receive, generating coordinating programs between specialized medical care and primary care, towards improving the care these patients receive. Currently, these warning systems are not done by systematic, nor automatic identification, and they are not extended to all Andalusian Health Service (SSPA). Furthermore, the use of the intraclass correlation coefficient (ICC) is recommended to quantify the reliability of the clinical measurements of the quantitative variables, either by repeating the measurement with the same instrument under the same conditions, or by determining the concordance of the valuations of the different instruments or observers under the same conditions [46].

When analyzing the overall concordance of the sample of polypathological patients, we noted that the concordance by categories is good or very good in all of the categories, except for G and H, where the concordance is moderate or weak. These results coincide with the description of the global cohort concordance mentioned above.

When analyzing the factors associated with a good concordance between the PP identification through the ICD-9-CM method and the clinical identification by agreement, coherent results were obtained. If the patient presented many clinical categories according to two or more observers, a greater association with a good concordance existed. On the other hand, the clinical categories associated with a good concordance are those that most often occur in polypathological patients, in this sense, heart, pulmonary, renal, and cerebrovascular diseases are highlighted. It is also reasonable that the more diagnoses presented at discharge, categorized by ICD-9-CM, the more likely the patient is to be polypathological, as they accumulate more diagnoses and more admissions than a complex patient with a high frailty and readmission.

This paper fundamentally reviewed the concepts that determine the validity of a test (sensitivity and specificity) and its accuracy (positive and negative predictive values), as well as the accuracy or probability that the test result predicts the presence or absence of a disease correctly. When evaluating any diagnostic test, it is essential to consider the same intrinsic properties, such as sensitivity and specificity. For its part, predictive values demonstrate a greater applicability in clinical practice, as they determined the usefulness of the test for the diagnosis of certain pathologies [47].

According to these results, we note that the proposed test presents very high sensitivity and specificity, which gives a good intrinsic validity, also by showing very high predictive values. For all of this, it can be stated that this is a safe test that can be applied in clinical practice. The accuracy has been very good, so the test is confirmed to accurately predict what was sought. All of this allows for stating that the identification of polypathological patients can be done automatically through ICD-9-CM coding as a population screening method, as the results of the validity and accuracy tests prove.

## 5. Conclusions

The clinical identification of polypathological patients and the automatic identification through ICD-9-CM coding obtained a good concordance strength. This leads to confirmthe possibility of using the automated identification of polypathological patients through ICD-9-CM coding as a population screening method. Its sensitivity, specificity, positive-, negative predictive values, and accuracy, with respect to clinical identification as a reference test, were very good. This supports its future use as a population screening method in health databases.

## Figures and Tables

**Table 1:** Defining clinical categories of polypathological patients (PP) and ICD-9-CM codes.

Clinical Categories		ICD-9 Codes
**A**		
A.1. Heart failure	Heart failure	428
	Long-term effect following cardiac surgery	429.4
	Hypertensive heart disease	402.91–402.01–402.11–404
A.2. Ischemic heart disease	Rheumatic heart failure (congestive)	398.91
	Ischemic heart disease	410 to 414
**B**		
B.1.Vasculitis and systemic autoimmune diseases	Diffuse diseases of connective tissue (systemic lupus erythematosus, RA (rheumatoid arthritis), Scleroderma, diffuse fasciitis, polymyositis, Sjogren, MCTD (mixed connective tissue disease)).	710
B.2. Chronic kidney disease	Polyarteritis nodosa and allied conditions	446
	Rheumatoid arthritis	714
	Polymyalgia rheumatica	725
	Chronic kidney disease	585
	Hypertensive chronic kidney disease	403
	Atherosclerosis of the renal artery	440.1
**C**		
Chronic airway obstruction, bronchial asthma or alveolar hypoventilation with functional limitation.	Chronic obstructive pulmonary disease	491 to 496–518.0
Chronic pulmonary heart disease	Acute and chronic respiratory failure	518.1
	Chronic pulmonary heart disease (unspecified)	518.83–518.84
		416.9
**D**		
D.1. Chronic inflammatory bowel disease	Inflammatory bowel disease	555–556
D.2. Syntomatic chronic liver disease or in activity	Chronic liver disease and cirrhosis (except for fatty liver and acute alcoholic)	571 (except for 571.0 and 571.1)
**E**		
E.1. Cerebrovascular disease	Cerebrovascular disease	430 to 438
	Other cerebral degenerations	331
E.2. Neurological disease with motor deficiency generating disability	Parkinson’s disease	332
	Other degenerative diseases of the basal ganglia	333
	Huntington chorea	333.4
	Torsion dystonia	333.6–333.7
E.3. Neurological disease with permanent cognitive impairment, at least moderate	Spinocerebellar disease	334
	Anterior horn cell disease	335
	Syringomyelia	336
	Multiple sclerosis	340
	Other demyelinating diseases	341
	Hemiplegia and hemiparesis	342
	Cerebral palsy	343
	Other paralytic syndromes	344
	Muscular dystrophies and other myopathies	359
	Senile dementia	290
	Other alcoholic dementia	291.2
	Dementia in conditions classified elsewhere	294.1
**F**		
F.1. Symptomatic peripheral vascular disease	Symptomatic peripheral vascular disease	443 (except for 443.81)
	Atherosclerosis of native arteries of the extremities	440.2
F.2. Diabetes mellitus with proliferative retinopathy or symptomatic neuropathy	Generalized atherosclerosis	440.9
	Diabetes mellitus	250.6– 250.5–352.5–362.01–362.07
		−2117.7
**G**		
G.1. Chronic anaemia through digestive blood losing or acquired hematologic disease unsuitable for treatment with curative intent	Iron deficiency anaemia secondary to blood loss (chronic)—Myelodysplastic syndrome	280.0–280.9–238.72–238.73–238.74–238.75
	Primary malignant neoplasm	140 to 195
	Secondary malignant neoplasms and metastatic	196 to 198
G.2. Solid neoplasia or active hematologic neoplasia unsuitable for treatment with curative intent	Malignant neoplasm without specification of site	199
	Malignant neoplasm of lymphatic and hematopoietic tissue	200 to 208
	Neoplasms of uncertain behaviour	235 to 238
	Neoplasms of unspecified nature	239
	Except for chemotherapy and radiotherapy admission	V58
**H**		
Chronic osteoarticular disease with functional limitation	Arthropathy associated with Reiter’s disease	711.1
	Arthropathy in Behcet’s syndrome	711.2
	Arthropathy, gastrointestinal conditions	713.1
	Crystal arthropathies	712
	Psoriatic arthropathy	696
	Arthropathy associated with hypersensitivity reaction	713.6
	Schönlein	713.5
	Arthropathy associated with neurological disorders	713.7
	Other general diseases with articular involvement	715
	Osteoarthrosis generalized	720
	Ankylosing spondylitis	

**Table 2:** Concordance between the PP classification according to clinical criteria and ICD-9-CM codes by categories.

Categories	Kappa	CI	Global Agreement Proportion	CI
A_1_ Heart failure	0.620 *	0.554–0.688	0.813	0.777–0.845
A_2_ Ischemic heart disease	0.794 *	0.741–0.846	0.902	0.873–0.925
A	0.655 *	0.587–0.723	0.845	0.811–0.873
B_1_ Vasculitis and systemic autoimmune diseases	0.751 *	0.617–0.884	0.976	0.958–0.986
B_2_ Chronic kidney disease	0.834 *	0.784–0.884	0.928	0.902–0.947
B	0.807 *	0.755–0.859	0.911	0.883–0.933
C Chronic airway obstruction, bronchial asthma, or alveolar hypoventilation with functional limitationChronic pulmonary heart disease	0.814 *	0.765–0.864	0.909	0.881–0.931
D_1_ Chronic inflammatory bowel disease	0.664 *	0.201–1.00	0.996	0.985–0.999
D_2_ Symptomatic chronic liver disease or in activity	0.798 *	0.706–0.889	0.966	0.947–0.979
D	0.805	0.716–0.893	0.966	0.947–0.979
E_1_ Cerebrovascular disease	0.525 *	0.443–0.608	0.811	0.775–0.843
E_2_ Neurological disease with motor deficiency generating disability	0.144 *	0.00–0.410	0.931	0.906–0.950
E_3_ Neurological disease with permanent cognitive impairment, at least moderate	0.568 *	0.44–0.691	0.918	0.891–0.939
E	0.568 *	0.503–0.647	0.802	0.766–0.834
F_1_ Symptomatic peripheral vascular disease	0.692 *	0.580–0.803	0.948	0.925–0.964
F_2_ Diabetes mellitus with proliferative retinopathy or symptomatic neuropathy	0.754 *	0.632–0.877	0.972	0.953–0.983
F	0.740 *	0.665–0.824	0.937	0.912–0.955
G_1_ Chronic anemia through digestive blood losing or acquired hematologic disease unsuitable for treatment with curative intent	0.490 *	0.321–0.658	0.939	0.914–0.957
G_2_ Solid neoplasia or active hematologic neoplasia unsuitable for treatment with curative intent	0.725 *	0.634–0.816	0.913	0.897–0.926
G	0.624 *	0.536–0.713	0.887	0.857–0.912
H Chronic osteoarticular disease with functional limitation	0.340 *	0.040–0.639	0.966	0.947–0.979

* *p* < 0.0001. PP—polypathological patient; CI—confidence interval.

**Table 3:** Factors associated with the correct identification of PP using ICD-9-CM coding (with respect to the gold standard clinical identification method).

Factors	Odds Ratio (β Exp)	CI 95%	*p*
Number of diagnoses grouped ICD-9-CM	0.928	0.889–0.969	0.001
Number of PP categories according to clinical criteria	0.590	0.445–0.782	0.000
C category according to clinical identification	1.807	1.158–2.820	0.009
E category according to clinical identification	1.752	1.199–2.559	0.004
E_3_ subcategory according to clinical identification	2.981	1.517–5.857	0.002
A_1_ subcategory according to clinical identification	2.042	1.000–4.167	0.05
A_2_ subcategory according to clinical identification	2.924	1.448–5.904	0.003
B_2_ subcategory according to clinical identification	2.208	1.311–3.718	0.003
F_2_ subcategory according to clinical identification	3.136	1.038–9.474	0.043

The reference category for every OR was the correct identification of PP using ICD-9CM.

**Table 4:** Sensitivity, specificity, positive predictive value (PPV), and negative predictive value (NPV) of identification of polypathological patients through ICD-9-CM with repsect to gold-standard clinical identification.

	ICD-9-CM (CI 95%)	ICD-9-CM without Category H (CI 95%)
**Sensitivity**	78.4% (74.8%–81.7%)	78.2% (74.5%–81.5%)
**Specificity**	87.8% (85.6%–89.7%)	88.2% (86.1%–90.1%)
**PPV**	78.1% (74.5%–81.4%)	78.6% (75.0%–81.9%)
**NPV**	88.0% (85.8%–89.9%)	88.0% (85.8%–89.8%)
**Accuracy**	84.5% (82.5%–86.2%)	84.7% (82.8%–86.4%)
